# CDC Trioplex diagnostic assay underperforms in detection of circulating Chikungunya West African genotype

**DOI:** 10.1128/jcm.00405-24

**Published:** 2024-06-13

**Authors:** Mignane Ndiaye, Mouhamed Kane, Diamilatou Balde, Safietou Sankhé, Maimouna Mbanne, Seynabou Mbaye Souna Diop, Umar Ahmad, Gerald Mboowa, Samba Niang Sagne, Mamadou Cisse, Ndongo Dia, Amadou Alpha Sall, Ousmane Faye, Gamou Fall, Oumar Faye, Manfred Weidmann, Moussa Moïse Diagne, Idrissa Dieng

**Affiliations:** 1 Virology Unit, Institut Pasteur de Dakar, Dakar, Senegal; 2 Africa Centres for Disease Control and Prevention (Africa CDC), Addis Ababa, Ethiopia; 3 Epidemiology Data Sciences and Clinical Research, Institut Pasteur de Dakar, Dakar, Senegal; 4 Institute of Microbiology and Virology, Brandenburg Medical School, Senftenberg, Germany; The University of North Carolina at Chapel Hill School of Medicine, Chapel Hill, North Carolina, USA

**Keywords:** Chikungunya virus, West African genotype, real time PCR, performance, CDC Trioplex RT-qPCR

## LETTER

Since 2015, the virology laboratory of the Institut Pasteur de Dakar (IPD) has been performing real-time molecular diagnostics of suspected arboviruses, including Chikungunya fever (CF) cases for the syndromic sentinel surveillance program of fevers 4S network (1). During the unprecedented recent largest ever CF outbreak in late 2023 in the southern part of the country affecting the regions of Kédougou and Tambacounda on October 2023, 210 RT-qPCR confirmed cases have been recorded (2). Due to the swift evolution of RNA viruses, it is essential to monitor mutations occurring within the primer- and probe-binding sites which may impact on the efficacy of these assays (3).

In response to the spread of Chikungunya virus (CHIKV) in Gambia (4) and in Burkina Faso (2) and the potential occurrence of an unprecedented number of CF cases in the subregion, we evaluated the performance of the donated CDC Trioplex RT-qPCR detection system for regular use, which allows the simultaneous detection of Zika virus, Dengue virus, and CHIKV using a set of serum samples collected during the initial phase of the ongoing epidemic in Senegal.

**Fig 1:**
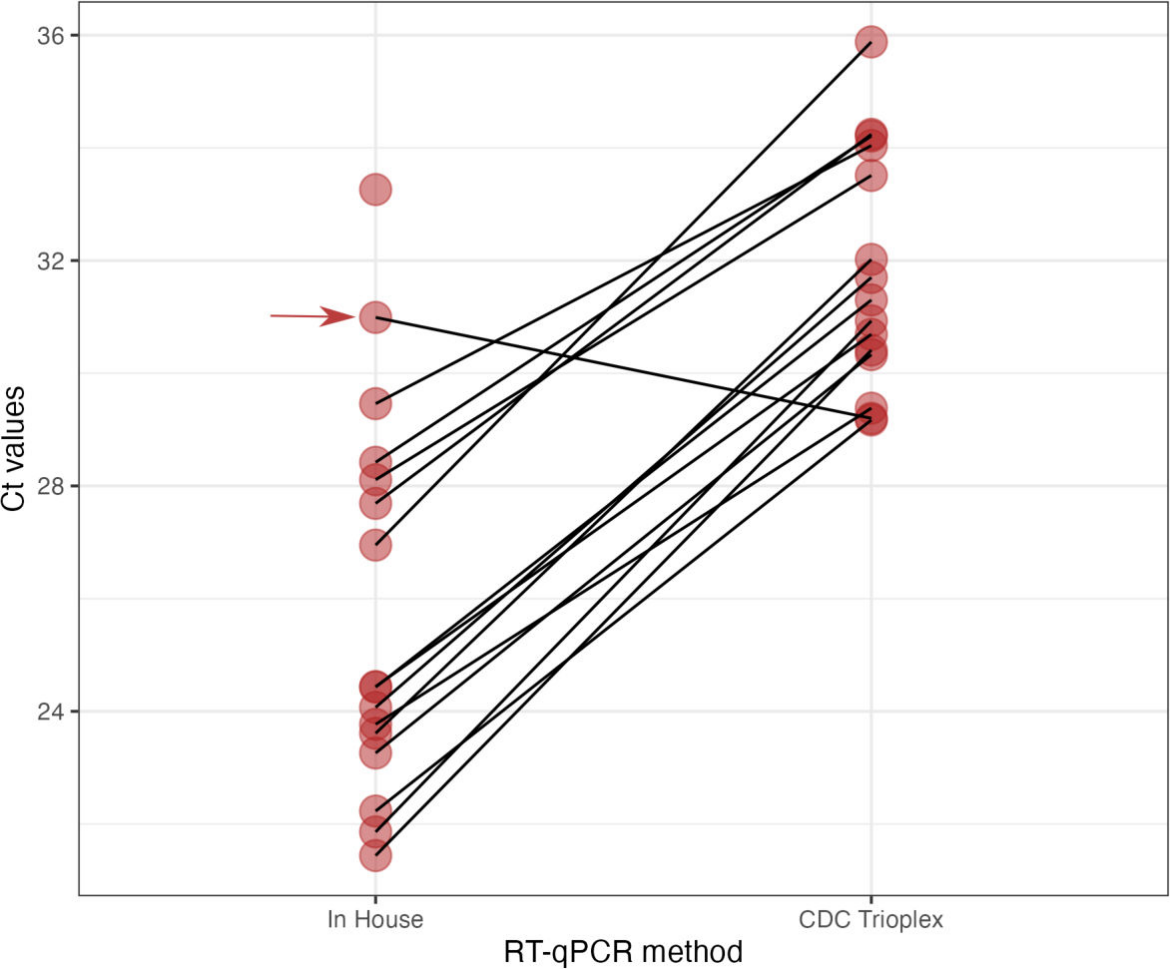
Comparison of Ct values for CHIKV between IPD in-house CHIKV PCR and CDC Trioplex PCR. On average, a delta CT value of 7 was observed. The CDC Trioplex kit positive control strain was the only RNA detected with a positive delta of CT 1.79 by the Trioplex CHIKV PCR (red arrow).

Surprisingly, we noticed a mean 7 Ct value delay of the Ct values of the CDC Trioplex in comparison to our in-house CHIKV RT-qPCR assay (5, 6) for 15 CHIKV RNA-positive samples collected from the ongoing Senegalese outbreak (Table S1; Fig. 1). To monitor for signature erosion, we performed whole genome sequencing of the RNA samples as described (7) (accession numbers in Table S1), and carried out an *in silico* analysis of both the in-house and CDC Trioplex oligonucleotides against targeted viral regions.

**Fig 2:**
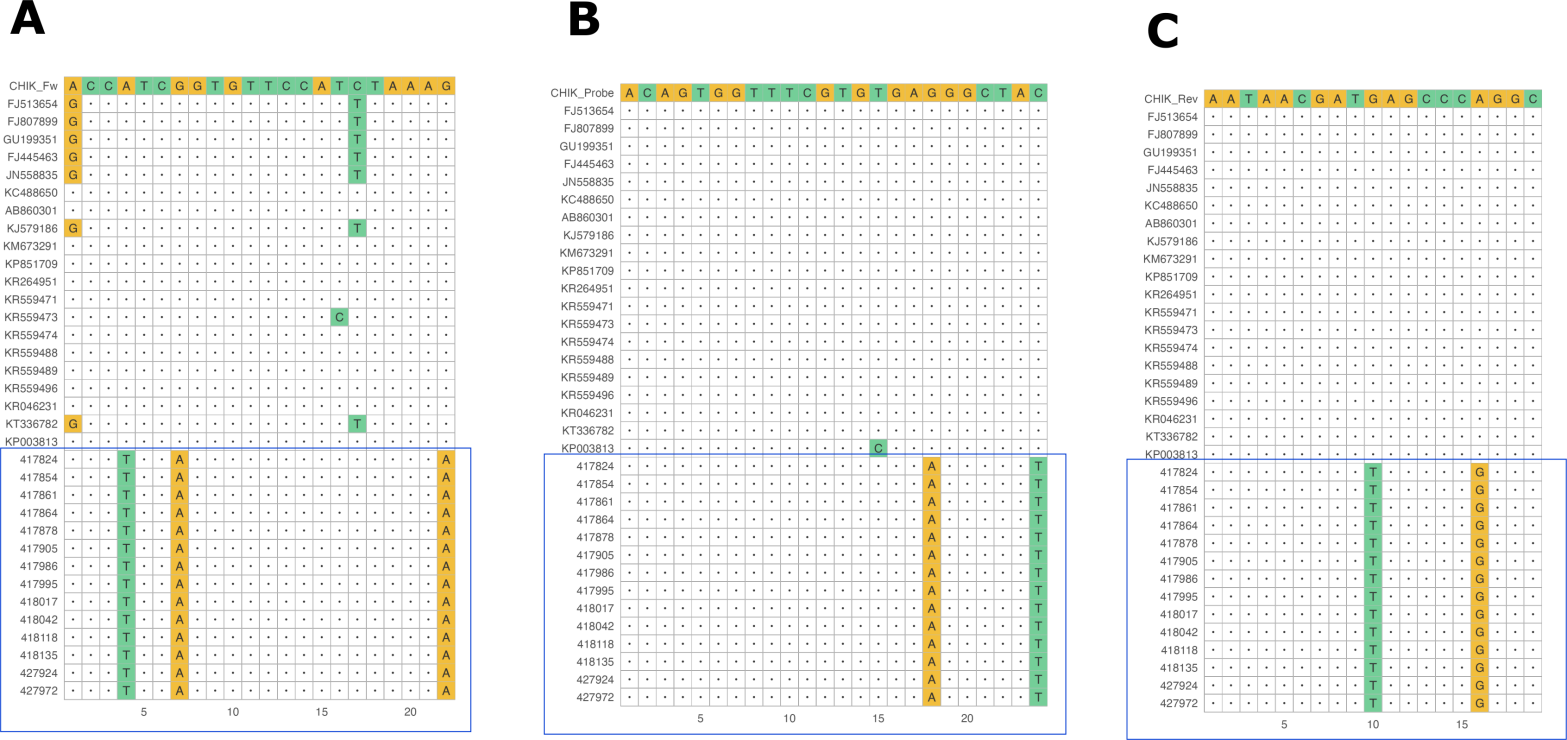
CHIKV Trioplex oligonucleotide against newly sequenced CHIKV WA genotype sequence NSP1 target sites highlighted in blue rectangle; A, B, and C indicate forward, probe, and reverse oligonucleotide, respectively.

The *in silico* analysis revealed three, two, and two mismatches in the target sequences of the circulating CHIKV West Africa (WA) genotype for the forward, the reverse, and the probe oligonucleotides, respectively (Fig. 2; Fig. S1). The most prominent mismatch is on the 3-prime end of the forward oligonucleotide, which is known to upset efficient elongation (8).

Additionally, previous studies show that genetic variation in the viral genome at primer/probe binding regions can result in potential mismatches and false negative results (9). Altogether, a total of seven mismatches across the CHIKV NSP1 amplicon including two in the probe have a significant impact on the performance of the Trioplex CHIKV PCR.

The detection shift of approximately two logs in quantity can potentially increase the false negative rate when using the CDC Trioplex PCR in samples with intermediate to low viral load (Ct values > Ct 28). The CHIKV WA genotype circulating in Senegal can still be detected by the IPD in-house assay (4). The *in silico* analysis revealed no mismatches of this oligonucleotide set when aligned to a broad range of previously described and newly determined contemporary CHIKV WA genotype sequences (Fig. S2). The only RNA detected earlier than the CHIKV WA RNA by the Trioplex assay is the RNA provided with the kit. The details of the origin of this RNA should be made available to allow verifying its sequence in alignments. It is most likely that the failure of the test is due to not including a West-African genotype strain in the validation panel for the Trioplex assay. These findings underscore the importance of monitoring assay performance and conducting genomic surveillance during outbreaks.

## Data Availability

Generated sequences during this work were deposed to GenBank with accession numbers PP236742 to PP236756.

## SUPPLEMENTAL MATERIAL

Supplemental material jcm.00405-24-s0001.docx Table S1; Figures S1 and S2.

ASM does not own the copyrights to Supplemental Material that may be linked to, or accessed through, an article. The authors have granted ASM a non-exclusive, world-wide license to publish the Supplemental Material files. Please contact the corresponding author directly for reuse.
