# Supplementary material for: CDC Trioplex diagnostic assay underperforms in detection of circulating Chikungunya West African genotype
Source: J Clin Microbiol. 2024 Jun 13;62(7):e00405-24. doi: 10.1128/jcm.00405-24 (PMC11250485; doi:10.1128/jcm.00405-24)
Supplement: Supplemental material — Table S1; Figures S1 and S2. [file jcm.00405-24-s0001.docx]

**CDC Trioplex Diagnostic Assay underperforms in detection of circulating Chikungunya West African genotype**

Mignane Ndiaye^1^, Mouhamed Kane^1^, Diamilatou Balde^1^, Safietou Sankhé^1^, Maimouna Mbanne^1^, Seynabou Mbaye Souna Diop^1^, Umar Ahmad^2^, Gerald Mboowa^2^, Samba Niang Sagne^3^, Mamadou Cisse^3^, Ndongo Dia^1^, Amadou Alpha Sall^1^, Ousmane Faye^1^,Gamou Fall^1^, Oumar Faye^1^, Manfred Weidmann^4^, Moussa Moïse Diagne^1^, Idrissa Dieng^1*^

^1^ Virology Unit, Institut Pasteur de Dakar

^2^ Africa Centres for Disease Control and Prevention (Africa CDC)

^3^ Epidemiology Data Sciences and Clinical Research, Institut Pasteur de Dakar

^4^ Institute of Microbiology and Virology, Brandenburg Medical School, Germany

**^*^Correspondence:** [idrissa.dieng@pasteur.sn](mailto:idrissa.dieng@pasteur.sn)

**Keywords:** Chikungunya virus, West African Genotype, real time PCR, performance, CDC trioplex RT-qPCR

**Supplementary files**

**Table S1 :** Ct values of PCR tests for 25 tested samples. ** Total RNA from CHIKV positive control strain included in the CDC trioplex kit.

| Sample ID | Collection Date | Collection site | Ct value in house | Ct value trioplex | Accession number |
| --- | --- | --- | --- | --- | --- |
| 427924 | 13.10.2023 | Matam | 21.44 | 30.41 | PP236755 |
| 418042 | 12.09.2023 | Kédougou | 21.86 | 30.93 | PP236752 |
| 417986 | 29.08.2023 | Kédougou | 22.23 | 29.17 | PP236748 |
| 417854 | 07.08.2023 | Kédougou | 23.26 | 30.33 | PP236743 |
| 418135 | 20.09.2023 | Kédougou | 23.61 | 32.02 | PP236754 |
| 417864 | 08.08.2023 | Kédougou | 23.77 | 29.38 | PP236745 |
| 418017 | 02.09.2023 | Kédougou | 24.07 | 31.7 | PP236751 |
| 417861 | 07.08.2023 | Kédougou | 24.43 | 31.3 | PP236744 |
| 417995 | 30.08.2023 | Kédougou | 24.44 | 30.69 | PP236749 |
| 418118 | 09.09.2023 | Kédougou | 26.95 | 35.88 | PP236753 |
| 417905 | 07.08.2023 | Kédougou | 27.69 | 34.24 | PP236747 |
| 417824 | 31.07.2023 | Kédougou | 28.11 | 33.51 | PP236742 |
| 417878 | 04.08.2023 | Kédougou | 28.42 | 34.24 | PP236746 |
| 427972 | 18.10.2023 | Matam | 29.46 | 34.04 | PP236756 |
| PC CHIKV** |  |  | 30.99 | 29.20 |  |


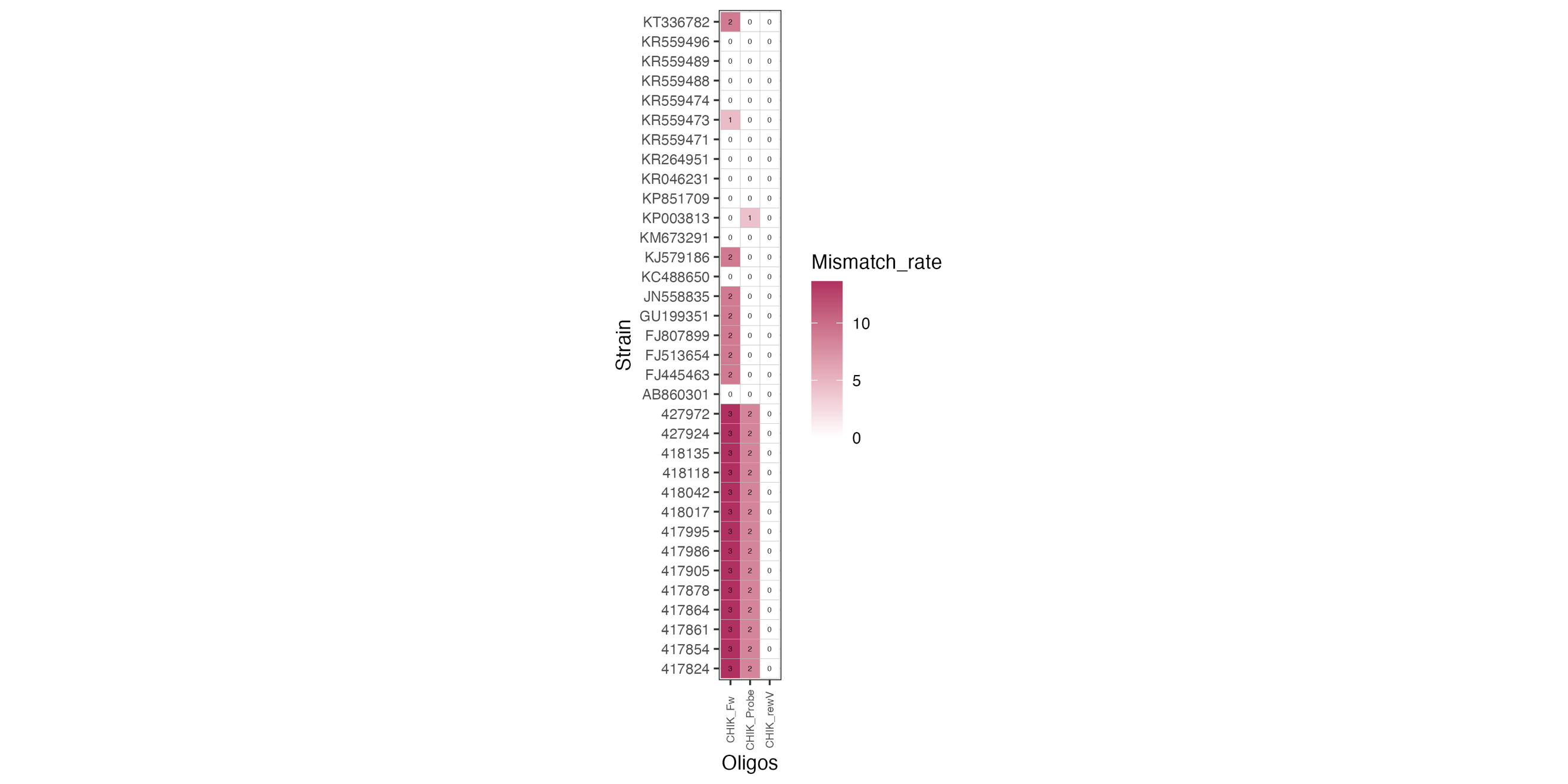


None West African

Genotype strains

**Figure S1.** Heatmap showing mismatches of trioplex CHIKV oligonucleotides in CHIKV sequences analysed. The number inside each box defines the number of observed mutations in the target sequences of the indicated CHIKV sequences.


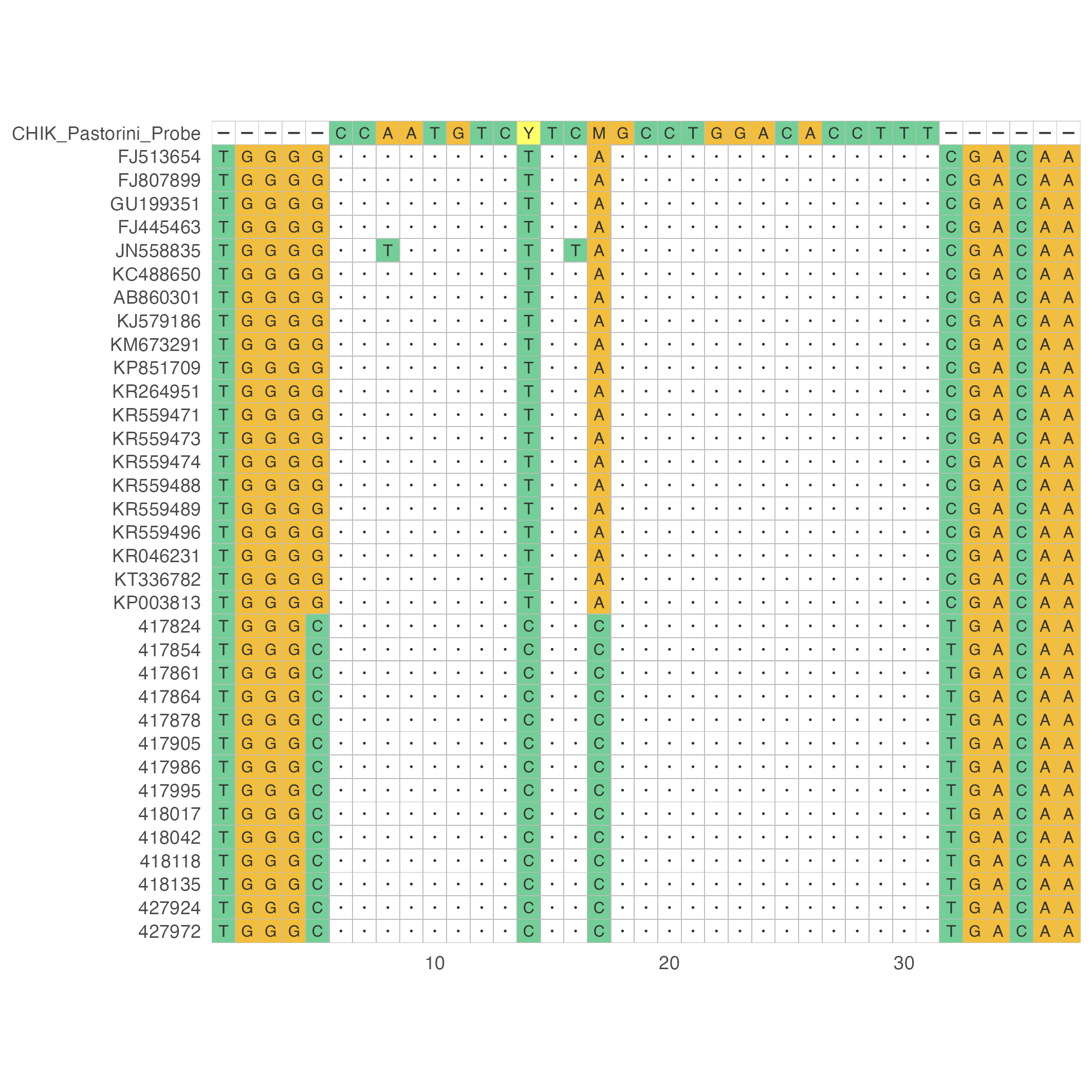

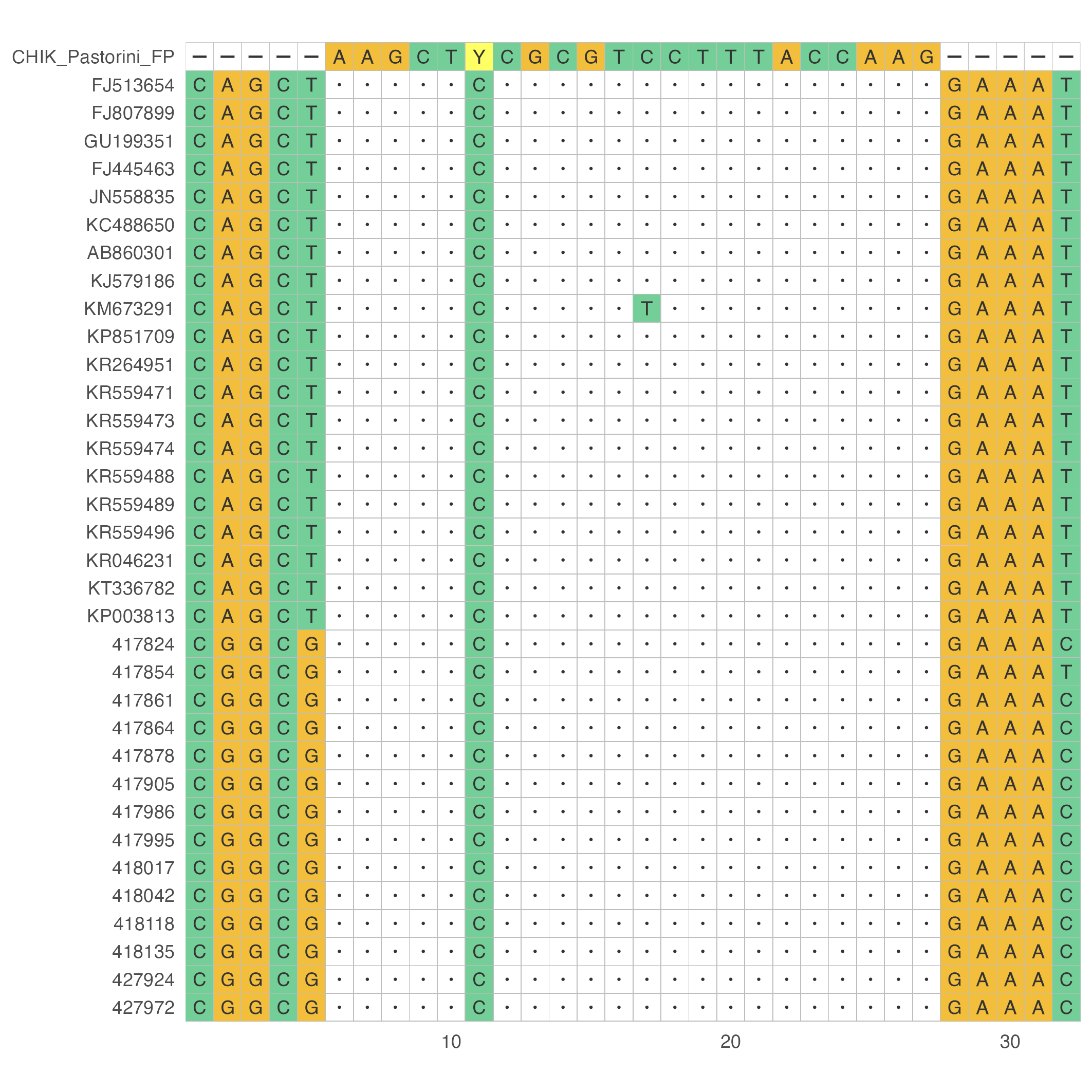

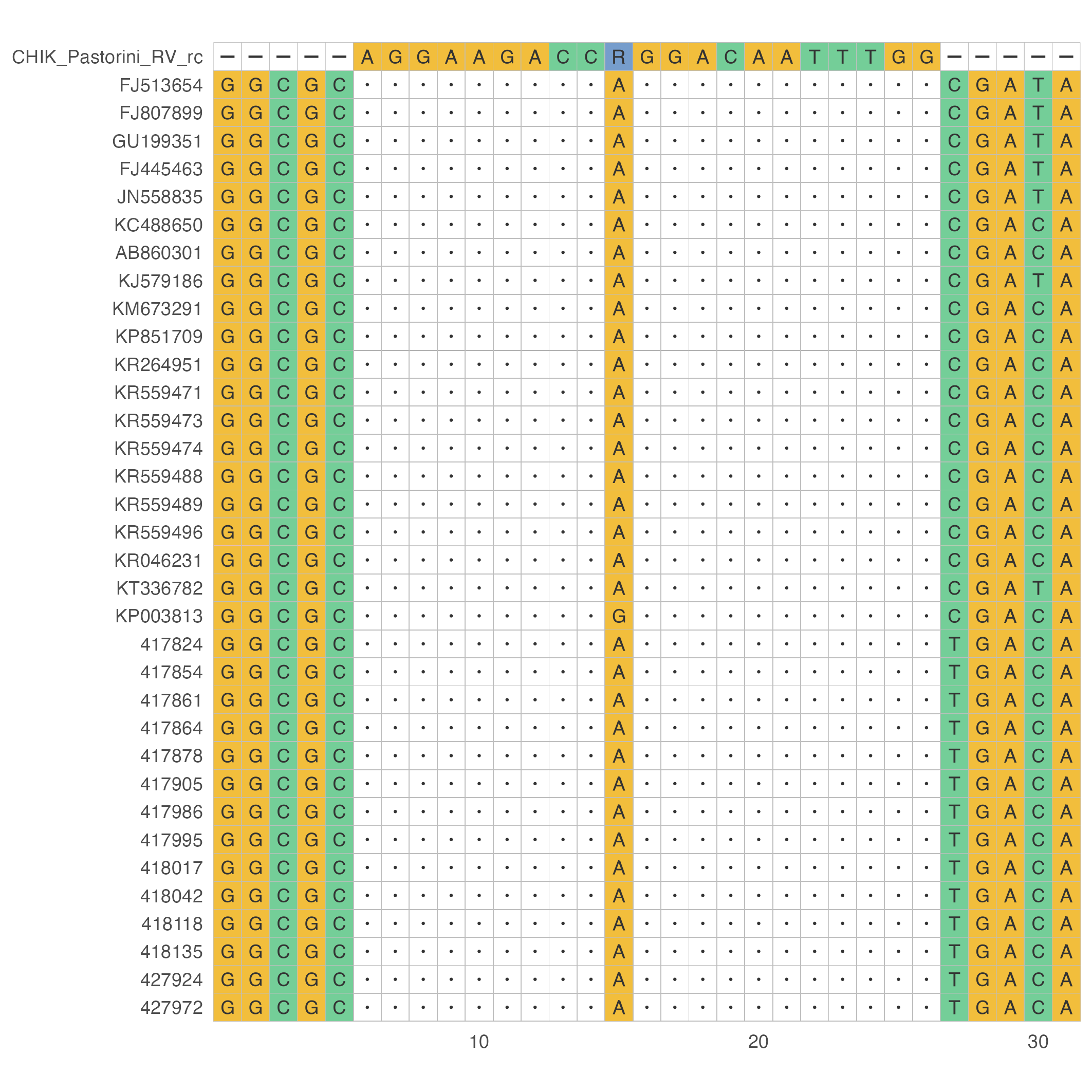


**Figure S2.** CHIKV Pastorino oligonucleotides against newly sequenced CHIKV WA genotype sequence E1 target sites highlighted in blue rectangle; A, B and C indicate forward, probe and reverse, oligonucleotide respectively.
